# Long-term spatiotemporal patterns in the number of colonies and honey production in Mexico

**DOI:** 10.1038/s41598-022-25469-8

**Published:** 2023-01-18

**Authors:** Francisco J. Balvino-Olvera, Jorge A. Lobo, María J. Aguilar-Aguilar, Gloria Ruiz-Guzmán, Antonio González-Rodríguez, Ilse Ruiz-Mercado, Adrián Ghilardi, María del Coro Arizmendi, Mauricio Quesada

**Affiliations:** 1 Laboratorio Nacional de Análisis y Síntesis Ecológica, Escuela Nacional de Estudios Superiores, Unidad Morelia, 58190 Morelia, Michoacán México; 2 Universidad de Costa Rica, Escuela de Biología, San Pedro, 2600 Costa Rica; 3 Instituto de Investigaciones en Ecosistemas y Sustentabilidad, Universidad Nacional Autónoma de México, 58190 Morelia, Michoacán México; 4 Escuela Nacional de Estudios Superiores, Unidad Mérida, Tablaje Catastral N°6998, Carretera Mérida-Tetiz Km. 4.5, 97357 Ucú, Yucatán México; 5 Centro de Investigaciones en Geografía Ambiental, Universidad Nacional Autónoma de México, 58190 Morelia, Michoacán México; 6 Laboratorio de Ecología, Unidad de Biotecnología y Prototipos (UBIPRO), Facultad de Estudios Superiores Iztacala Universidad Nacional Autónoma de México, Av. de los Barrios 1, Los Reyes Iztacala, 54090 Tlalnepantla, Estado de México México

**Keywords:** Climate-change impacts, Agroecology, Ecology, Ecology, Environmental sciences, Environmental social sciences

## Abstract

Honey bee decline is currently one of the world's most serious environmental issues, and scientists, governments, and producers have generated interest in understanding its causes and consequences in honey production and food supply. Mexico is one of the world’s top honey producers, however, the honey bee population's status has not been documented to date. Based on 32 years of data from beekeeping, we make a country-level assessment of honey bee colony trends in Mexico. We use generalized additive mixed models to measure the associations between the percent change in honey bee hives and the percent change in honey yield per hive in relation to land-use, climate, and socioeconomic conditions. Despite the fact that the average annual yield per hive increased from 1980 to 2012, we detected a significant decline in the percent change in the number of honey bee hives across the time period studied. We also found a relationship between climatic conditions and agricultural land use, with agriculture increases and high temperatures producing a decrease in the percent change in honey yield. We found a relationship between a reduction in the temperature range (the difference between maximum and minimum temperatures) and a decrease in the percent change in the number of hives, while socioeconomic factors related to poverty levels have an impact on the number of hives and honey yields. Although long-term declines in hive numbers are not correlated with poverty levels, socioeconomic factors in states with high and medium poverty levels limit the increase in honey yield per hive. These results provide evidence that land-use changes, unfavorable climatic conditions, political, and socioeconomic factors are partially responsible for the reductions in the percent change in honey bee hives in Mexico.

## Introduction

*Apis mellifera* L., the European honey bee, is one of the most widely managed insects in the world and one of the most common flower visitors in both natural and agricultural landscapes^1^. As a result of their role in maintaining plant diversity, food production assurance, and honey production; honeybee populations are one of the best studied insect systems^2–4^. Unfortunately, local, national, and regional losses in the number of managed honey bee colonies have been documented but these trends vary greatly between regions. Data on *Apis mellifera* populations and production is critical for detecting reductions in the number of honey bee hives^5^. Geographic biases in research efforts create gaps in our understanding of the scope, degree, and causes of the reduction in the number of honey bees worldwide. For example, there are declines in colonies for North America and Europe^6,7^; but there are apparent increases for Asia, Africa, and South America^8–10^. Latin America, encompasses some of the largest producers and exporters of honey worldwide; despite this, no conclusive subcontinental data on colony losses has been released to date^11^. Recent regional research reveals that parasites like the *Varroa destuctor* mite, as well as non-biological hazards like agriculture intensification, queen failure, pesticide use, and nutritional deficits, are the most prominent problems affecting honey bees in Latin America^12–15^. Moreover, the FAO (Food and Agriculture Organization) data suggest a stagnation of honey production relative to worldwide trends, as well as high rates of colony losses in the region^10^. Mexico is the world's fifth-largest exporter of honey and Mexican beekeeping has significant social and economic implications with roughly 43,000 beekeepers producing around 61,000 tons of honey yearly, making Mexico the world's fifth-largest exporter of honey^16^. Furthermore, due to its quality Mexican honey is in high demand on the international market, with a commercial value of honey production estimated at 67.9 USD million dollars each year^16^. Relative to other countries in Latin America, Mexico is a well-studied region with respect to long-term agricultural and honey production monitoring^17^. Nonetheless, assessing the population status of managed honeybee colonies in Mexico is a difficult task for several reasons: first, it is a highly diverse country that allows beekeeping to be practiced in a wide range of climatic conditions and habitats; second, economic heterogeneity encourages the existence of beekeepers with a wide range of technological and production capabilities^11,18^. Therefore, Mexico represents a challenging country to understand the reduction in the number of honey bee hives, but it is imperative to document the impact of the reduction in the number of honey bee hives and its potential relationship to the ecosystem services provided to this tropical nation. Information from the Honey Bee Colony Mortality Monitoring Program (COLOSS) revealed that several parts of Mexico face high winter loss risks^19–21^. These previous studies on Mexican beekeeping, on the other hand, have focused on short temporal datasets, which result in insufficient sample size, with limited scope, and the inability to investigate dynamic spatiotemporal fluctuations. As shown by national surveys in the US, overwintering losses had no discernible impact on the total managed colony numbers, perhaps as a result of beekeepers' ability to promptly replace losses^22^. The lack of data at sufficiently large spatiotemporal scales that can capture links between changes in honey bee hive populations and long-term environmental changes is partly to blame for the little attention paid to analyzing the integrative effects of climate, socioeconomic, and land use changes on beekeeping. To assess whether the problem of reductions in the number of honey bee hives is a phenomenon that affects Mexican beekeeping activity, we analyze 32 years of government available datasets on this activity nationwide^16^. It has been difficult to establish a causal factor for the decline in honey bee population, but pesticides, climate change, landscape alterations, socioeconomic factors, water stress, and agricultural intensification and their interactions could have all been responsible^23–25^. Beekeepers in México began to document significant colony losses where adult bees abruptly disappeared from hives virtually all at once. This phenomenon was initially related to Colony Collapse Disorder (CCD) which was first reported in the United States in 2006^26^. In this study, we examine historical patterns in bee production and bee populations in Mexico. The first objective of our study was to examine some of the key drivers to explain changes in the number of hives and honey production including, agricultural land use, climate, and socioeconomic factors. A second objective of the study was to describe the space–time variability in colony losses at the national level. We are particularly interested in recognizing spatiotemporal patterns in hive trends that suggest local decline effects. Understanding the factors that drive honey bee population trends, as well as the direction and degree of changes, is critical for the timely adoption of management practices, prevention measures, and ultimately, risk factor mitigation.

## Results

**Figure 1:**
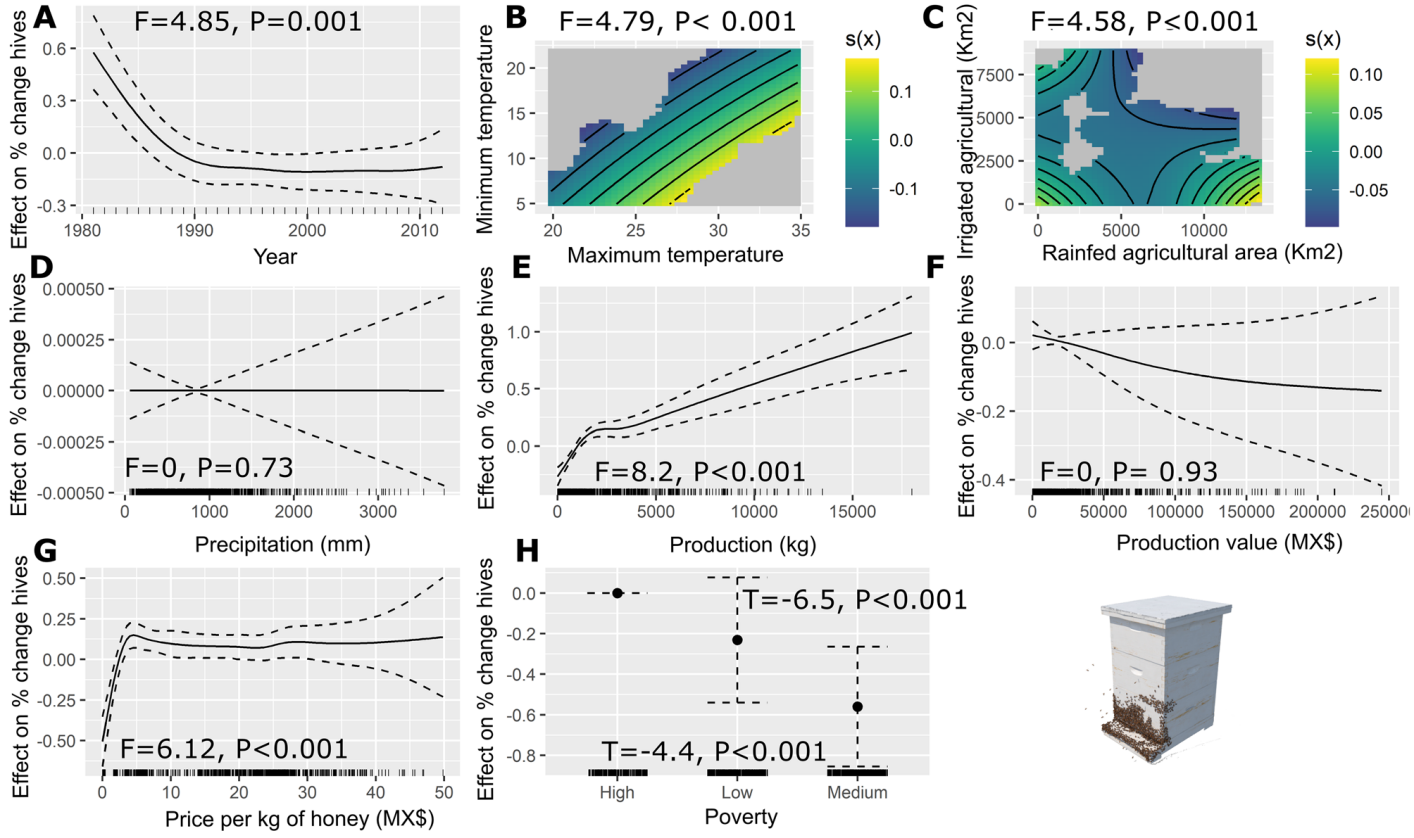
Estimated smoothness of; (**a**) year, (**b**) non-linear interaction between minimum temperature and maximum temperature, (**c**) non-linear interaction between land use categories, (**d**) precipitation (mm), (**e**) production of honey (kg), (**f**) production value (MX$), (**g**) price per kg of honey (MX$), and (**h**) poverty over the percent change in honey bee hives; y-axis is the partial effect of the variable and the shaded area represents 95% of the confidence interval around the main effect.

In this study, we calculated the rate of change per year in the number of hives and the honey yield from 1980 to 2012 for Mexico. The response variables analyzed are the percent change in the number of hives and the percentage change in yield, where negative values indicate losses and positive values indicate increases. Over the sampled period from 1980 to 2012, the estimated percent change in honey bee hives significantly decreased over time (F = 4.85, edf = 4.3, P < 0.001) (Fig. 1A). The tensor product smooth between minimum temperature and maximum temperature variables has a significant effect (F = 4.79, edf = 1.1, P < 0.001) on the percent change in honey bee hives. We found a positive relationship between the increase in temperature ranges (i.e. difference between the maximum temperature and minimum temperature) and an increase in percent change in the number of hives (Fig. 1B). The tensor product smooth between agricultural land use variables show that when the area of irrigated agriculture and rainfed agriculture increases, the percentage change in the number of hives decreases (F = 4.58, edf = 1.1, P < 0.001) (Fig. 1C). We found no significant effect of the precipitation on the percent change in the number of hives (F = 0, edf = 3.7, P = 0.73) (Fig. 1D). The results of the GAMMs model, indicate a positive correlation between honey production and an increase in the percent change in the number of hives (F = 8.2, edf = 4.3, P < 0.001) (Fig. 1E). We found no correlation between the value of honey production and the percentage change in the number of hives (F = 0, edf = 4.8, P = 0.93) (Fig. 1F). We found a positive relationship between the price of honey per kilogram with an increase in the percent change in the number of hives (Fig. 1G) (F = 6.12, edf = 8.3, P < 0.001). The percentage change in the number of hives exhibits, on average, significant negative rates of change in the states with medium (T = − 6.5, P < 0.001) and low (T = − 4.4, P < 0.001) poverty (Fig. 1H). There is no significant percent change in the number of hives in the states with high poverty.

**Figure 2:**
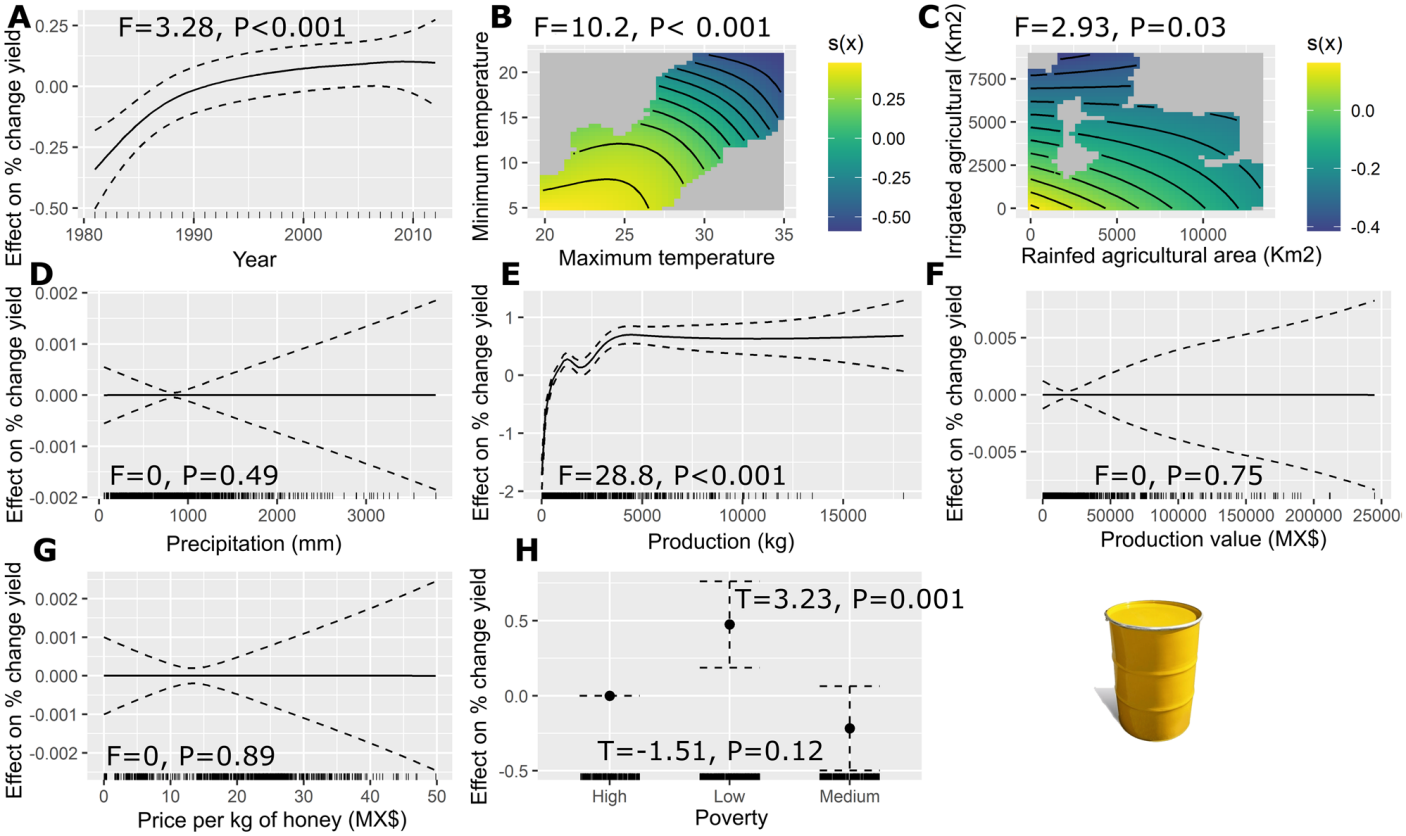
Estimated smoothness of; (**a**) year, (**b**) non-linear interaction between minimum temperature and maximum temperature, (**c**) non-linear interaction between land use categories, (**d**) precipitation (mm), (**e**) production of honey (kg), (**f**) Production value (MX$), (**g**) price per kg of honey, and (**h**) Poverty over the percent change in yield; y-axis is the partial effect of the variable and the shaded area represents 95% of the confidence interval around the main effect.

GAMMs analyses also showed a positive relationship between the year and the percentage change in yield (F = 3.28, edf = 2.4, P < 0.001) (Fig. 2A). The tensor product smooth between minimum temperature and maximum temperature variables has a significant negative effect (F = 10.2, edf = 4.9, P < 0.001) on the percent change in yield, with high temperatures resulting in low yields (Fig. 2B). Similarly, the tensor product smooth between irrigated and rainfed agricultural area had a significant negative effect (F = 2.93, edf = 3, P = 0.03) on the percent change in yield, with high values in the agricultural area causing negative rates in the percent change in yield (Fig. 2C). We found no correlation between the precipitation and the percentage change in yield (F = 0, edf = 2.6, P = 0.49) (Fig. 2D). The results of the GAMMs model, also show a significant effect of the percentage change in yield over the total production of honey per year (F = 28.8, edf = 8.3, P < 0.001), with positive rates of percent change in honey yield favoring high production values (Fig. 2E). We found no correlation between the value of honey production (F = 0, edf = 3.7, P = 0.75), the price per kg of honey (F = 0, edf = 5.4, P = 0.89) and the percentage change in yield (Fig. 2F,G). Regarding the proportion of the population living in poverty per state, we found that states with low poverty increased percent rates of change in yield (T = 3.23, P = 0.001), and states with medium and high poverty showed no significant changes in the percent change in yield (T = − 1.51, P = 0.12) (Fig. 2H).

**Figure 3:**
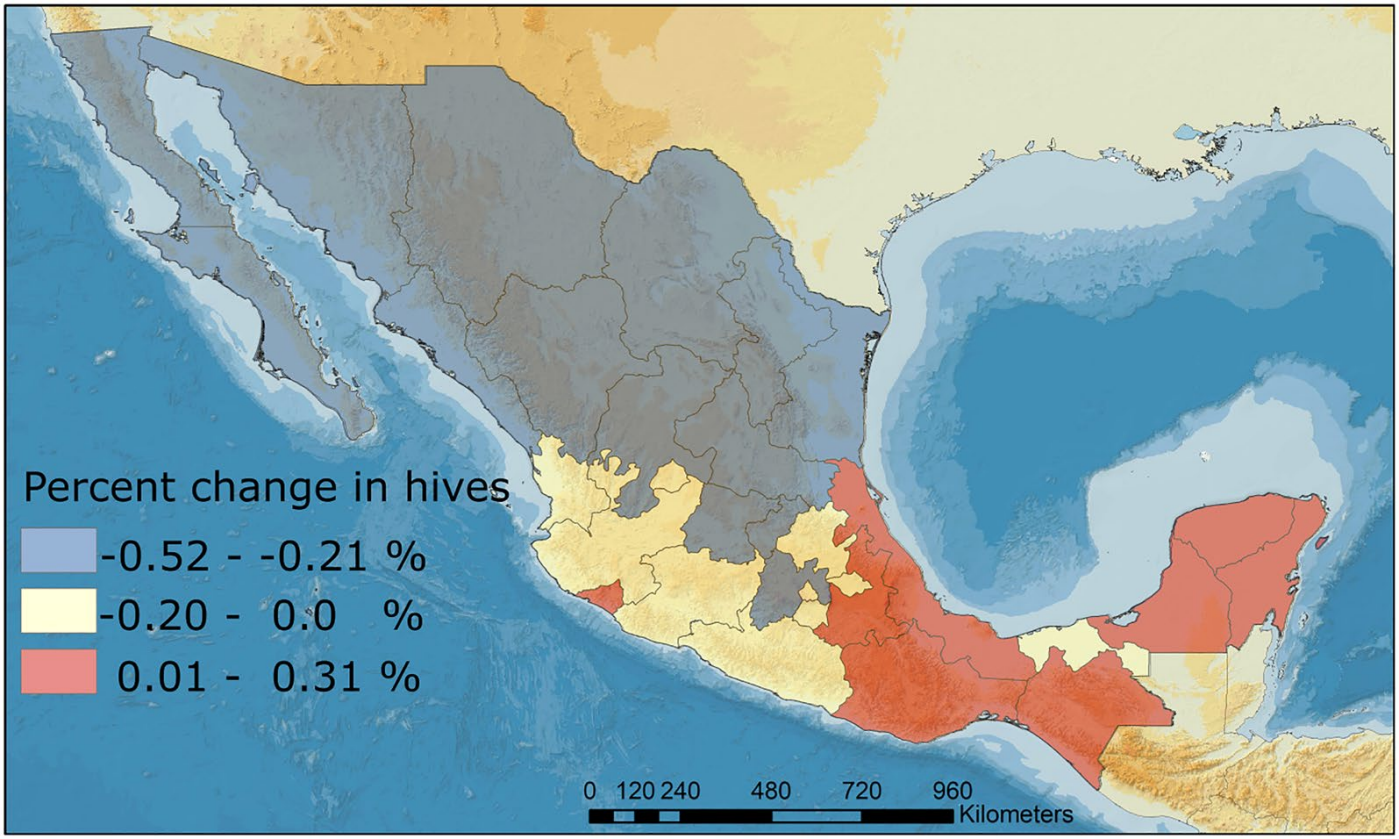
Predicted spatial patterns from the GAMM model for the percent change in honey bee hives between 1980 and 2012 across Mexico.

**Figure 4:**
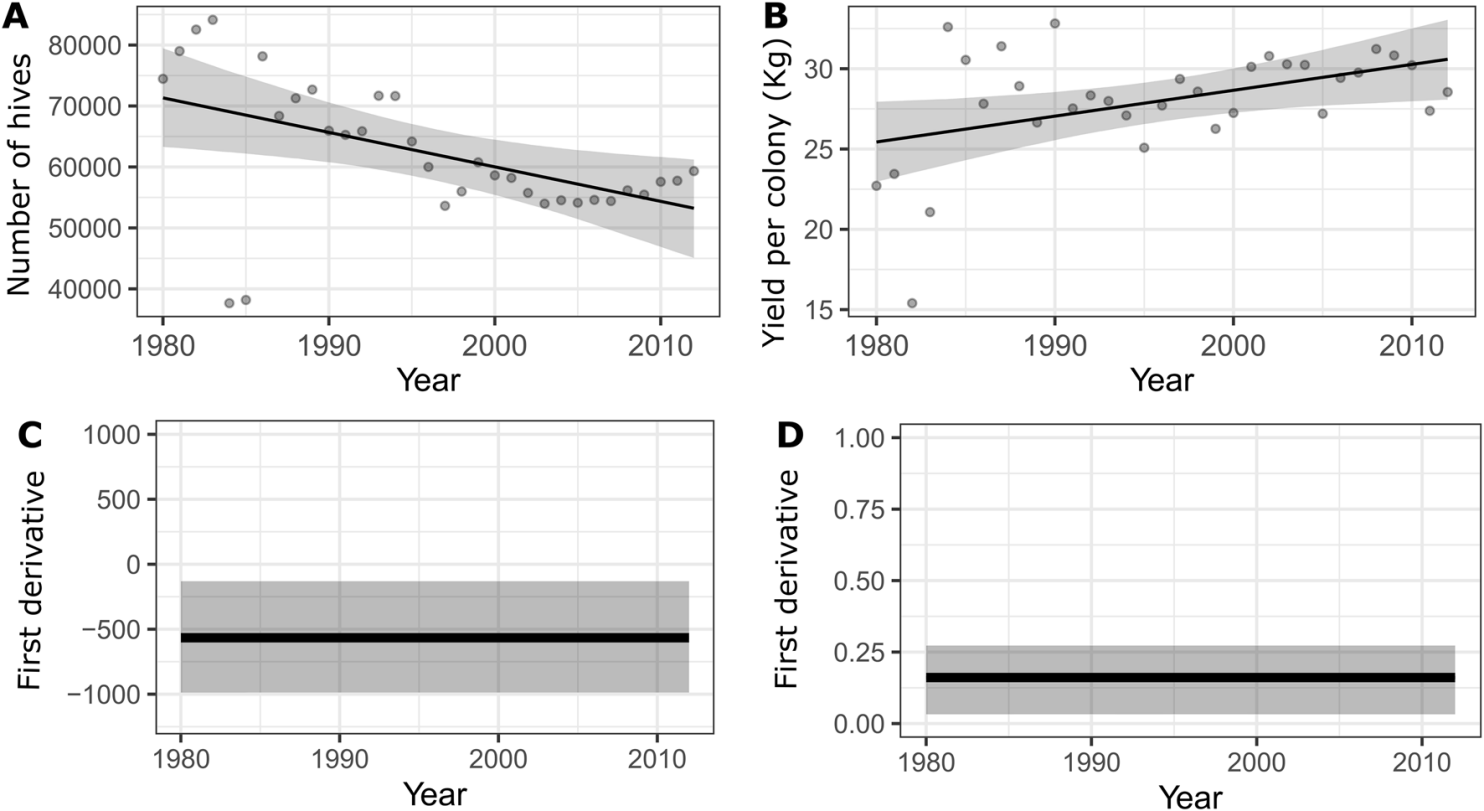
The estimated national average trend for: (**a**) the number of hives and (**b**) the average annual yield per hive (kg). The first derivative is estimated for the slope of (**c**) the number of hives and (**d**) the average annual yield per hive. The shaded area represents approximately 95% of the confidence intervals. The sections of the slope where the confidence interval does not include zero are indicated by the sections of the trend line in black.

**Figure 5:**
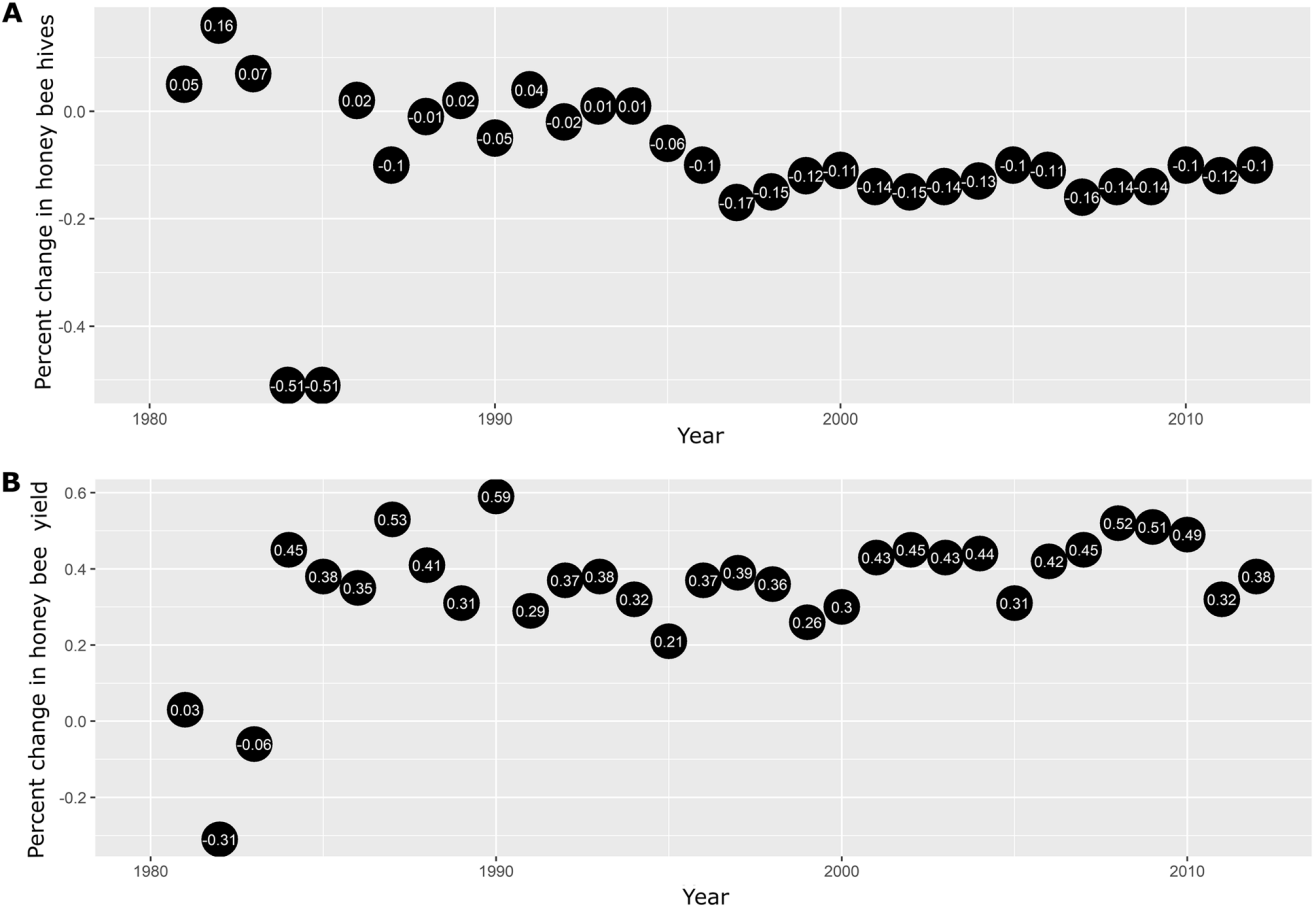
Temporal variations in the percentage change in the number of hives in Mexico based on the population in 1980.

Long-term trends in the percent change in the number of hives in each Mexican state are highly variable, but they provide useful geographical information on the behavior of managed honeybee populations at a regional level. Figure 3 depicts the long-term trends in the number of hives according to the best fit model for the percent change in honey bee hives (Table 1), in which states in dark brown showed an increase and the rest of the states gradually decrease. The central section (states of Estado de Mexico, Queretaro, Guanajuato) and north (states of Baja California, Chihuahua, Coahuila, Sonora, Durango, San Luis Potosi, Tamaulipas, and Nuevo Leon) are areas of Mexico with significantly high rates of reductions in the number of hives (− 0.21% to − 0.52%). The central-western region, which comprises the states of Nayarit, Jalisco, Michoacan, and Guerrero, and states as Mexico City, Morelos, Hidalgo, Aguascalientes, and Tabasco, maintains or experiences reductions in the percent change in the number of hives of less than − 0.2%. In contrast, the southern Mexican states of Yucatan, Campeche, Quintana Roo, Chiapas, Veracruz, Oaxaca, Puebla, and Colima exhibit significant increases in the percent change in the number of hives (0.01–0.31%) (Fig. 3). According to the national average in the number of hives, there was a significant and continuous reduction in honey bee colonies between 1980 and 2012 (Figs. 4A,C, and 5A). Furthermore, since the beginning of bee production monitoring, the average honey yield has increased significantly (Figs. 4B,D, 5B).

**Table 1:** The GAMM analysis results for percent change in yield, and percent change in the number of honey bee hives, including covariates for each model.

Best model	Variable	Estimate	e.d.f	s.e	t-value	F-value	p-value	r^2^	n
	Intercept	0.32		0.88	3.7		< 0.001		
	Poverty low	− 0.46		0.10	− 4.44		< 0.001		
	Poverty medium	− 0.65		0.10	− 6.57		< 0.001		
	Production (kg)		4.36			8.20	< 0.001		
	Production value		4.84			0	0.93		
	Price (kg)		8.33			6.12	< 0.001		
	Year		4.34			4.85	< 0.001		
	T_max_-T_min_		3.87			4.79	< 0.001		
	Rainfed area: irrigated area		1.17			4.58	< 0.001		
	Precipitation		3.72			0	0.73		
Percent change in number of hives	Percent change ~ s(Production kg, bs = "cs") + s(Production value, bs = "cs") + s(Price per kg, bs = "cs") + s(Year, bs = "cs") + te(Tmax, Tmin, bs = "ps") + te(Rainfed agricultural area km^2^, Irrigated crop area km^2^, bs = "ps") + s(Precipitation mm., bs = "cs") + %Poverty, family = Gamma(link = "log"), correlation = corAR1(State))							0.327	965
	Intercept	0.23		0.11	1.95		0.05		
	Poverty low	0.47		0.14	3.23		0.001		
	Poverty medium	− 0.21		0.14	− 1.51		0.12		
	Production (kg)		8.32			28.8	< 0.001		
	Production value		3.77			0	0.75		
	Price (kg)		5.47			0	0.89		
	Year		2.45			3.28	< 0.001		
	T_max_-T_min_		4.98			10.23	< 0.001		
	Rainfed area: irrigated area		3.0			2.93	0.03		
	Precipitation		2.64			0	0.49		
Percent change in yield	Percent yield ~ s(Production kg, bs = "cs") + s(Production value, bs = "cs") + s(Price per kg, bs = "cs") + s(Year, bs = "cs") + te(Tmax, Tmin, bs = "ps") + te(Rainfed agricultural area km^2^, Irrigated crop area km^2^, bs = "ps") + s(Precipitation mm., bs = "cs") + %Poverty, family = Gamma(link = "log"), correlation = corAR1(State))							0.102	965

*e.d.f* estimated degrees of freedom, *s.e* standard error.

## Discussion

Mexico is one the largest honey producers globally, however, relatively little is known about the honey bee colony trends and the status of *A. mellifera* populations nationwide*.* The purpose of this research was to examine some of the most important drivers that may influence the number of hives and honey production including agricultural land use, climate, and socioeconomic drivers as well as their potential impact on honeybee population. Our study showed that honey bee colonies declined significantly at the national level (Figs. 4A,C, and 5A), and a downward trend in the number of hives has been observed in nearly half of Mexico's states (Figs. S1–S3; Table S1). The percent change in yield of honey per hive increased significantly, reaching a national annual average of 28.9 kg per hive (Figs. 4B,D, and 5B). This growth is most likely due to beekeeping modernization and high-cost management practices. Osterman et al. (2021) discovered that, as a result of a rising in per capita honey demand, honey production per colony has increased by 33% globally in the last seven decades^9^. Between 1986 and 1993, one of the most significant insect invasion events associated with Africanized honey bee migration from Brazil to North America was linked to honey bee losses and a reduction in honey production in Mexico because many beekeepers abandoned the activity due to apparent difficulties in managing these bee hives^27^. Even though beekeeping has become more complex due to the colonies' highly defensive behavior, there is no conclusive evidence between reduction in the number of colonies, honey production, and Africanization under all environmental conditions present in Mexico^28,29^. Similar to other countries where Africanization occurred, in Mexico, honey production has partially rebounded and remained relatively stable despite the fact that the Mexican beekeeping sector has faced new threats like the parasite mite *V. destructor* discovered in 1992^30^. Conversely, multiple reasons including pesticide exposure and agricultural intensification appear to be influencing the reductions in the number of managed honeybee colonies^24,31,32^.

Meta-analyses on pollinator decline, demonstrated that the interaction between climate change and intensive agricultural land use is linked to a nearly 50% decline in insect abundances and 27% in the number of insect species^33^. Bees, being ectothermic organisms with a short life cycle, generally respond quickly to environmental changes in precipitation and temperature^34^. The European bee, *A. mellifera*, requires temperature conditions between 34 and 35 °C for the proper development of the larvae, so temperature and environmental humidity represent the most important abiotic factors that determine the development of the larvae and behavior of the colonies, and weather plays a significant role in determining temperature and humidity^35,36^. According to our research, the tensor product smooth between the minimum and maximum temperature has a relationship in which the decrease in the temperature range (the difference between the maximum and minimum temperatures) reduces the percent change in the number of hives. There are several different aspects of climate change that can be studied at various temporal and spatial scales, including alterations in boundaries (maxima and minima), average conditions, and variance. An examination of global temperature changes over the past 50 years reveals a significant reduction in temperature ranges^37,38^. The reduction in temperature ranges, resulting from minimum temperatures warming faster than maximum temperatures, is an important indicator of shifts caused by climate change, it indicates whether the weather is stable or not. However, the effects on insects are poorly understood but may be severe, affecting their ability to recover from daytime heat stress and having indirect effects on plants^39^. Similarly, the temperature has a significant impact on the percentage change in yield. Our findings suggest that minimum temperatures above 15 °C and maximum temperatures over 32 °C have negative rates of percent change in yield. Foraging can take place in a wide temperature range, from 10 to 40 °C, outside the hive^40^. Bees limit their foraging trips at low temperatures, which typically is not below 6.5 °C with an optimum of 20 °C^41^. Consistent with our findings, experimental studies show that when daily average temperatures are around 17 °C, the trends in honey production are positive, but turn negative when temperatures rise above 17 °C, demonstrating a non-linear relationship between temperature and beehive productivity^42^. Other studies that examine long-term trends in honey production in response to environmental variables find a strong negative relationship between honey production and temperature. In particular, in Puerto Rico, honey yields decreased with greater temperature seasonality^43^. The negative effect of high temperatures is related to both a decrease in food availability (nectar, pollen, and honeydew) and changes in colony phenology and bee activity. It is thought that high humidity levels have an impact on the physical characteristics of pollen and the nectar's sugar content. Humidity and rain tend to have a negative effect on flying activity, making foraging more challenging. In addition, an increase in humidity and rain can reduce pollen availability, and dilute nectar production in flowers^44^. However, our findings did not support the existence of a correlation between precipitation and the percent change in the number of hives and yield. Beekeeping activities face formidable obstacles worldwide. The main threat to honey bees in the future is predicted to be climate change^45^. Climate change can impact honey bee colonies negatively and/or positively, but in tropical regions, climate change has the potential to reduce yields almost by half^43^. According to our findings, temperature range reductions are related with low yields, implying that under climate change scenarios, a rise in world temperatures (and therefore a reduction in the temperature ranges) can have a considerable impact on beekeeping production in Mexico.

In agricultural landscapes, pollinators' health also depends on the types of agricultural practices because of differences in the exposure to toxins, and the quality and quantity of food sources^32,46^. Two broad categories can be distinguished in the Mexican agricultural industry. On the one hand, subsistence or traditional agriculture, usually polyculture (milpas), relies on rain (i.e. rainfed agriculture), minimal agricultural inputs use, low use of pesticides, and low-salaried labor to produce crops. On the other hand, commercial or industrial agriculture increases productivity by using monocultures, large amounts of fertilizers and pesticides, and highly mechanized agriculture. Although irrigated or industrial agriculture modality in Mexico covers four times less area than rainfed agriculture^47^, we find a strong correlation between increases in irrigated industrial agricultural area with respect to declines in the percent change in yield. Historical trends in agricultural land use indicate that irrigated industrial agriculture area has shown increases in most of the country, but specifically in the North of Mexico which has a strong impact on reducing honey bee colonies^47,48^. A possible explanation for the relationship between honey bee declines and the increases of both categories of agricultural land use, but particularly of irrigated industrial cropland, is attributable to agricultural expansion that, in turn, reduces natural habitats decreasing the availability of floral resources in natural areas and increasing nutritional deficits^49^. Pollen and nectar provide all the energy and nutrients necessary for honey bee colonies, so access to a variety of floral plant sources is critical for long-term colony survival. Monocultures frequently only supply floral resources for relatively short periods of time, resulting in honey bee nutritional stress, which may diminish immunocompetence and increase parasite loads^50,51^. In contrast, in non-industrial farming systems with a higher diversity of floral resources (i.e. poly-floral diet), phenological replacement occurs gradually, ensuring honey bees access to diverse and continuous food sources at all times^52^.

Around the world agricultural landscapes also have been shown to affect honey production, brood production, and honeybee colony survival^49,53,54^. However, large data sets on honey bee colony losses and agricultural land use has only been analyzed in a few studies with contrasting results^55,56^. Clermant et al. (2015), discovered that honey bee colony losses are linked to 60 of 133 land cover classes, with the majority of these classes being linked to human activities other than agriculture. In contrast, Kuchling et al. (2018) discovered that locations with a high amount of seminatural areas have a lower risk of colony losses when compared to areas with a high proportion of artificial surfaces, mainly agricultural environments. Modern agriculture increasingly relies on the use of chemical substances to control weeds, fungi, and arthropod pests to ensure high yields. Pesticides pose a risk to pollinators due to their toxicity and amount of exposure, which varies geographically depending on the compounds employed, as well as the extent to which land and habitat have been managed in the landscape. Spatiotemporal data on pesticide use in agriculture provide additional information about honey bee colony dynamics^57^. Unfortunately, Mexico lacks official or regulatory statistics on the number of pesticides used, the frequency with which they are applied, or their use on specific crops. Consequently, is necessary to establish accurate statistics that quantify pesticide use at the national level. In Mexico, land cover use trends suggest that agricultural expansion is the most important driver of ecosystem change, in the present and future scenarios^58^. At the same time, researchers and beekeepers became concerned due to localized declines in beekeeping production and the discovery of highly harmful agricultural contaminants in samples of honey, pollen, and wax throughout Mexico at amounts that are very close to the lethal dose per bee^59–63^. In general, studies of the effect of pesticides on *A. mellifera* colonies in Mexico have shown negative effects on the behavior and health of this bee species^64–66^. The most studied compound (GF-120) can alter foraging behavior and is lethal in high amounts. Valdovinos-Nuñez et al., (2009) evaluated the toxicity of the pesticides Permethrin, Methomyl, Diazinon in wild bees *Melipona beecheii*, *Trigona nigra* and *Nannotrigona perilampoides* and found that all these species were very vulnerable to these chemicals, younger bees are more vulnerable than older bees, and males are more susceptible than females in *M. beecheii*^67^.

Other factors that affect beekeeping and reduce honey production include predators, parasites, and illnesses. Honey bees in Mexico, are vulnerable to bacterial diseases (such as American and European foulbrood), viral diseases (such as the Israeli Acute Paralysis Virus), fungal diseases (such as Nosema species), and parasitic infections (such as Varroa spp.)^68^. Despite being widely recognized as a significant factor by producers, hive loss caused by numerous parasites and viruses is a poorly studied in Mexico, lacking national and periodical disease incidence records. In addition, a recent study also reveals that the hive's water supply can be a critical factor in the hive's health. A historical review of data generated from experimental water supply studies revealed that the half-life of honey bees in the US has been reduced from 1970 to 2021 from an average of 34.3 days to 17.7 days, respectively^25^. Unfortunately, it is not possible to conduct a similar comparative study for Mexico due to a lack of data.

Long-term trends in the hive populations and honey production can also be impacted by political and economic factors^69,70^. Mexican honey is in high demand on the global market due to its quality, and its price per kilogram has risen over the research period^16^. Despite the fact that there is a decline in the number of hives and honey production nationwide, the value of honey seems to be more related to the market economy (offer and demand), rather than cost-effectiveness in beekeeping activities. Our findings show that in states with low and medium poverty, the percentage change in the number of hives is significantly lower, but not in states with high poverty, implying that the indices used to calculate poverty, such as per capita income, the average educational gap in the household, access to health services, access to social security, the quality and space of the housing, access to nutritious and quality food, and the degree of social cohesion, are not directly related to the long trends in the number of hives. For Mexico, the fact that states with low poverty have significantly positive rates of increase in the percent change in yield is mostly attributable to the influence of increased availability of infrastructure and production inputs. Therefore, implementing governmental initiatives for infrastructure development and technological innovation has the potential to increase productivity, particularly in states with medium and high poverty where yield have decreased over time according to our study.

Collectively, our results show that the North and Central regions of Mexico are the most vulnerable regions to reductions in the number of honey bee hives due to the convergence of extreme climatic conditions (droughts and low temperatures) and high rates of industrial agricultural expansion. Indeed, during the last years, the lagoon region of Coahuila-Durango, and the states of Tamaulipas, Sinaloa, and Sonora in the north region of Mexico are among the most recurrent areas with increases in the number of cases of sudden mortality of large numbers of hives, and our data support this pattern over the 32 years of our study period. More research is needed to identify the relative relevance of the drivers of the reduction in the number of hives, changes in agricultural development policies, the generation of infrastructure and technology innovation, and designing climate change mitigation measures are required to ensure food security and the long-term viability of beekeeping and pollinator services in México.

GAMMs analysis is a useful tool to estimate the variation in occurrence probability of bee species as a function of spatial and temporal variation in climate (minimum temperature, cumulative precipitation, maximum temperature), land-cover changes (proportion of habitats or human impacts within a buffer), and socioeconomic drivers. Future efforts should focus on similar, multidriver analyses on honey bee trend populations or honey bee colony losses worldwide, given that the diverse stressors operate differentially in distinct geographical regions. Quantifying honey bee responses to climate change, land degradation, parasites, and pesticides is a crucial step toward understanding the role that these factors may have played in long-term global colony declines.

## Conclusion

The spatiotemporal patterns and relative changes in honey production and colony losses were investigated using GAMMs analysis, which captured the nonlinear and heterogeneous effects of agricultural land use, climatic conditions, and socioeconomic factors. Even though data has been collected since 1980, this is the first study that provides spatiotemporal modeling of the beekeeping industry nationwide in Mexico. We found a considerable reduction in the percent change of honey bee hives across the study period, while the average honey production per hive increased from 1980 to 2012. We found that poverty is not directly related to the long trends in the number of hives, with states with low and medium poverty, decreasing in the percent change in the number of hives but not states with high poverty. Besides, we found that in states with high and medium poverty, socioeconomic factors limit the increase in honey yields per hive, making them less competitive and more subject to environmental conditions that affect yields in these regions. We found a strong correlation between climatic conditions and agricultural land use production systems, with industrial agriculture increases and high temperatures resulting in a decrease in percent change in honey yield. Furthermore, decreases in temperature ranges decrease the percent change in the number of hives. Our findings show the importance of land-use type, climatic conditions, and socioeconomic factors in honey bee production and population colony trends, emphasizing that pollinator-friendly agriculture practices, climate change mitigation, and government initiatives for the development of infrastructure should be taken into account in national pollinator and beekeeping development projects. Reduction in the number of honey bee hives must be addressed, anticipated, and mitigated to ensure the economic future and sustainability of pollination-dependent agriculture.

## Methods

### Data collection

The data for the analyses were compiled from two different public databases; agricultural censuses and honey productivity censuses were collected from the Agro-Food Information System of Consultation, by the Secretary of Agriculture and Rural Development (SADER) of the Government of Mexico for the period between 1980 and 2012^16^. Honey production is dependent on the weather and the availability of flower nectar, so in the majority of Mexico, it happens primarily during two seasons of the year. The first is produced between the months of March and May in the Southeast and Coastal regions of the nation (spring–summer). The second is achieved in the country's North and Altiplano between September and November (autumn–winter). In this study, the amount of honey produced (kg) over the two harvest seasons recorded for Mexico is used to determine hive productivity. Data on beekeeping include honey production value, number of hives, price per kilogram, and honey yield per hive (kg).

The agriculture sector in Mexico can be divided into two broad divisions due to the significant socioeconomic differences between these two groups. On the one hand, subsistence, or traditional agriculture, usually polyculture (milpas), relies on rain (i.e. rainfed agriculture), minimal agricultural inputs use, and low-salaried labor to produce crops. On the other hand, commercial or industrial agriculture increases productivity by using monocultures, large amounts of fertilizers and pesticides, and highly mechanized agriculture. The SADER database distinguishes these groups like irrigated and rainfed agriculture. Agricultural data included the total rainfed and irrigated cropland areas (km^2^) of 304 crop species produced in each of Mexico’s 32 states from 1980 to 2012.

Annual averages of climatic time series between 1980 and 2012 for each state were taken from the National Climatological Database^72^.

Finally, in order to use a proxy to understand how potential socioeconomic factors affect beekeepers in Mexico, we have included estimates of the percentage of the population living in poverty in each state from the National Council for the Evaluation of Social Development Policy (CONEVAL)^73^. From this information, we have generated three poverty categories that include: high (50–70% of the population), medium (35–50% of the population), and low (22–35% of the population of each state). Poverty in Mexico is estimated by taking into account the current per capita income, the average educational gap in the household, access to health services, access to social security, the quality and space of the housing, access to nutritious and quality food, the degree of social cohesion, and the degree of accessibility to paved roads.

### Statistical analyses

To investigate the first objective of the study which evaluates the impact of agricultural land use, climate, and socioeconomic drivers on honey bee production and colony trends, we use generalized additive mixed models (GAMMs) that allow nested data structures, and account for non-linear relationships between covariables and response variables^74,75^. We used the mgcv^74^ statistical package to perform the GAMMs statistical analyses in R version 3.4.2^76^. In order to determine the rates of change in the number of hives and the yield, we calculated the percentage change of both beekeeping variables by each Mexican state. During earlier data examination, we found autocorrelation in the time series of both the percent change in honey bee hives and the honey yield variables. Therefore, an autoregressive order 1 model (corAR1) was used to reduce the effect of spatial dependencies on the temporal trends^75^. In residual autocorrelation control, this approach produces reliable estimates and standard errors^77^. In order to minimize the overparametrization of the models, a Pearson correlation analysis was done before adjusting the regression models to analyze the link between honey bee data and response variables. Three climate variables, three agricultural use variables, and four socioeconomic variables are included in the analysis. Because the climatic parameters considered are highly correlated (r > 0.7, Fig. S4), we only used for the analysis the variables maximum temperature, minimum temperature, and precipitation that have been established as biologically relevant in *A. mellifera*^43,78^. Then, we used the package performance in the statistical program R to determine the appropriate distribution family error^79^. Models were validated by visually inspecting the graphical representations of residues vs. fitted values to ensure homoscedasticity and other normality assumptions (Figs. S5 and S6). The periods of significant percent change in the number of hives and average annual yield trends were identified from the models' uncertainty considering the simultaneous confidence intervals and the first derivative from the trends as proposed by Simpson (2018)^80^. The second objective of the study analyzed the space–time variability in colony losses at the national level. The tensor product interaction te(Latitude, Longitude) calculated as the centroids of the polygons in each Mexican state was integrated into the model and presented in the fields package of the statistical program R^81^. To visualize spatial trends in percentage change in honey bee hives, we plotted the values obtained from the GAMM model for the response variable for each state in ArcView 10.0.

## Supplementary Information

Supplementary Information.

## Data Availability

All data used in this publication are publicly accessible at https://www.gob.mx/siap/documentos/siacon-ng-161430, https://smn.conagua.gob.mx/es/climatologia/informacion-climatologica/informacion-estadistica-climatologica, and https://www.coneval.org.mx/Medicion/Paginas/PobrezaInicio.aspx.
