# Supplementary material for: Long-term spatiotemporal patterns in the number of colonies and honey production in Mexico
Source: Sci Rep. 2023 Jan 18;13:1017. doi: 10.1038/s41598-022-25469-8 (PMC9849204; doi:10.1038/s41598-022-25469-8)
Supplement: Supplementary file 1 — Supplementary Information. [file 41598_2022_25469_MOESM1_ESM.docx]

**Supplementary Information**

**Long-term spatiotemporal patterns in the number of colonies and honey production in Mexico**

Francisco J. Balvino-Olvera^1^, Jorge A. Lobo^2^, María J. Aguilar-Aguilar^1^, Gloria Ruiz-Guzmán^1^, Antonio González-Rodríguez^3^, Ilse Ruiz-Mercado^4^, Adrián Ghilardi^1,5^, María del Coro Arizmendi^6^ & Mauricio Quesada^1, 3,*^

^1^Laboratorio Nacional de Análisis y Síntesis Ecológica, Escuela Nacional de Estudios Superiores, Unidad Morelia, Morelia, Michoacán, 58190 México

^2^Universidad de Costa Rica, Escuela de Biología, San Pedro, 2600 Costa Rica

^3^Instituto de Investigaciones en Ecosistemas y Sustentabilidad, Universidad Nacional Autónoma de México, Morelia, Michoacán, 58190 México

^4^Escuela Nacional de Estudios Superiores, Unidad Mérida, Tablaje Catastral N°6998, Carretera Mérida-Tetiz Km. 4.5, Ucú, Yucatán, 97357 México.

^5^Centro de Investigaciones en Geografía Ambiental, Universidad Nacional Autónoma de México, Morelia, Michoacán, 58190 México

^6^Laboratorio de Ecología, Unidad de Biotecnología y Prototipos (UBIPRO), Facultad de Estudios Superiores Iztacala Universidad Nacional Autónoma de México. Av. de los Barrios 1, Los Reyes Iztacala, Tlalnepantla, Estado de México 54090, Mexico

* Mauricio Quesada; e-mail: mquesada@cieco.unam.mx

**Supplementary data**

**Table S1.** Summary table of linear regression analysis of the number of hives and year (1980-2019) for each Mexican republic state

| **Model summary** | | | | | **ANOVA** | | | | |
| --- | --- | --- | --- | --- | --- | --- | --- | --- | --- |
| Model | Estimate | R square | Ajusted  R square | Std. Error  of the  Estimate | Sum of squares | df | Mean square | F | P |
| Aguascalientes | 41.992 | 0.5364 | 0.5232 | 6.599 | 2262.5 | 1 | 2262.54 | 40.496 | < 0.001 |
| BCS | 46.91 | 0.4979 | 0.4836 | 28.811 | 2100.2 | 1 | 2100.22 | 34.71 | < 0.001 |
| CDMX | 33.451 | 0.281 | 0.2604 | 9.045 | 1185.1 | 1 | 1185.14 | 13.677 | < 0.001 |
| Chiapas | 37.452 | 0.5991 | 0.5877 | 5.178 | 2527.1 | 1 | 2527.1 | 52.308 | < 0.001 |
| Colima | -23.015 | 0.2458 | 0.2243 | 6.814 | 1037 | 1 | 1036.98 | 11.41 | < 0.001 |
| Estado de México | -34.884 | 0.5397 | 0.5265 | 5.446 | 2276.3 | 1 | 2276.34 | 41.033 | < 0.001 |
| Hidalgo | -40.752 | 0.6148 | 0.6038 | 5.453 | 2593.1 | 1 | 2593.1 | 55.855 | < 0.001 |
| Michoacán | -38.86 | 0.3911 | 0.3737 | 8.196 | 1649.7 | 1 | 1649.72 | 22.482 | < 0.001 |
| Nayarit | -44.055 | 0.6324 | 0.6219 | 5.677 | 2667.5 | 1 | 2667.5 | 60.216 | < 0.001 |
| Nuevo León | -44.688 | 0.6104 | 0.5992 | 6.035 | 2574.6 | 1 | 2574.57 | 54.831 | < 0.001 |
| Oaxaca | 52.07 | 0.3592 | 0.3409 | 11.75 | 1515.2 | 1 | 1515.19 | 19.621 | < 0.001 |
| Querétaro | -28.027 | 0.7883 | 0.7823 | 2.455 | 3325.1 | 1 | 3325.1 | 130.33 | < 0.001 |
| Sonora | -14.92 | 0.2882 | 0.2679 | 3.964 | 1215.8 | 1 | 1215.79 | 14.174 | < 0.001 |
| Tabasco | -27.272 | 0.4801 | 0.4653 | 4.797 | 2025.2 | 1 | 2025.23 | 32.326 | < 0.001 |
| Tamaulipas | -40.954 | 0.467 | 0.4517 | 7.396 | 1969.6 | 1 | 1969.64 | 30.661 | < 0.001 |
| Tlaxcala | 26.697 | 0.6029 | 0.5915 | 3.663 | 2542.9 | 1 | 2542.88 | 53.131 | < 0.001 |
| Veracruz | -59.44 | 0.2959 | 0.2758 | 15.5 | 1248.3 | 1 | 1248.28 | 14.712 | < 0.001 |
| Zacatecas | -52.87 | 0.3615 | 0.3433 | 11.88 | 1524.8 | 1 | 1524.84 | 19.817 | < 0.001 |
| Jalisco | -43.41 | 0.1842 | 0.1609 | 15.44 | 776.9 | 1 | 776.92 | 7.902 | 0.01 |
| BC | 33.45 | 0.1442 | 0.1175 | 14.4 | 551.2 | 1 | 551.17 | 5.394 | 0.05 |
| Chihuahua | 27.05 | 0.1181 | 0.09286 | 12.5 | 498 | 1 | 497.97 | 4.685 | 0.05 |
| Sinaloa | -23.125 | 0.1631 | 0.1392 | 8.853 | 688.1 | 1 | 688.09 | 6.8226 | 0.01 |
| Campeche | 7.277 | 0.005039 | -0.02339 | 17.283 | 21.3 | 1 | 21.256 | 0.1773 | 0.6 |
| Coahuila | 6.828 | 0.04039 | 0.01297 | 5.626 | 170.4 | 1 | 170.36 | 1.4731 | 0.2 |
| Durango | -7.305 | 0.0106 | -0.01767 | 11.931 | 44.7 | 1 | 44.698 | 0.3749 | 0.5 |
| Guanajuato | -21.63 | 0.06747 | 0.04083 | 13.59 | 284.6 | 1 | 284.59 | 2.5323 | 0.1 |
| Guerrero | 16.21 | 0.03318 | 0.005557 | 14.79 | 140 | 1 | 139.96 | 1.2012 | 0.2 |
| Morelos | -8.88 | 0.01484 | -0.01331 | 12.237 | 62.6 | 1 | 62.584 | 0.5271 | 0.4 |
| Puebla | 34.07 | 0.06028 | 0.03343 | 22.74 | 254.2 | 1 | 254.25 | 2.245 | 0.1 |
| Quintana Roo | -11.52 | 0.01256 | -0.01565 | 17.26 | 53 | 1 | 52.987 | 0.4453 | 0.5 |
| San Luis Potosí | -15.64 | 0.05256 | 0.02549 | 11.23 | 221.7 | 1 | 221.71 | 1.9418 | 0.1 |
| Yucatán | 4.503 | 0.002665 | -0.02583 | 14.726 | 11.2 | 1 | 11.241 | 0.0935 | 0.7 |


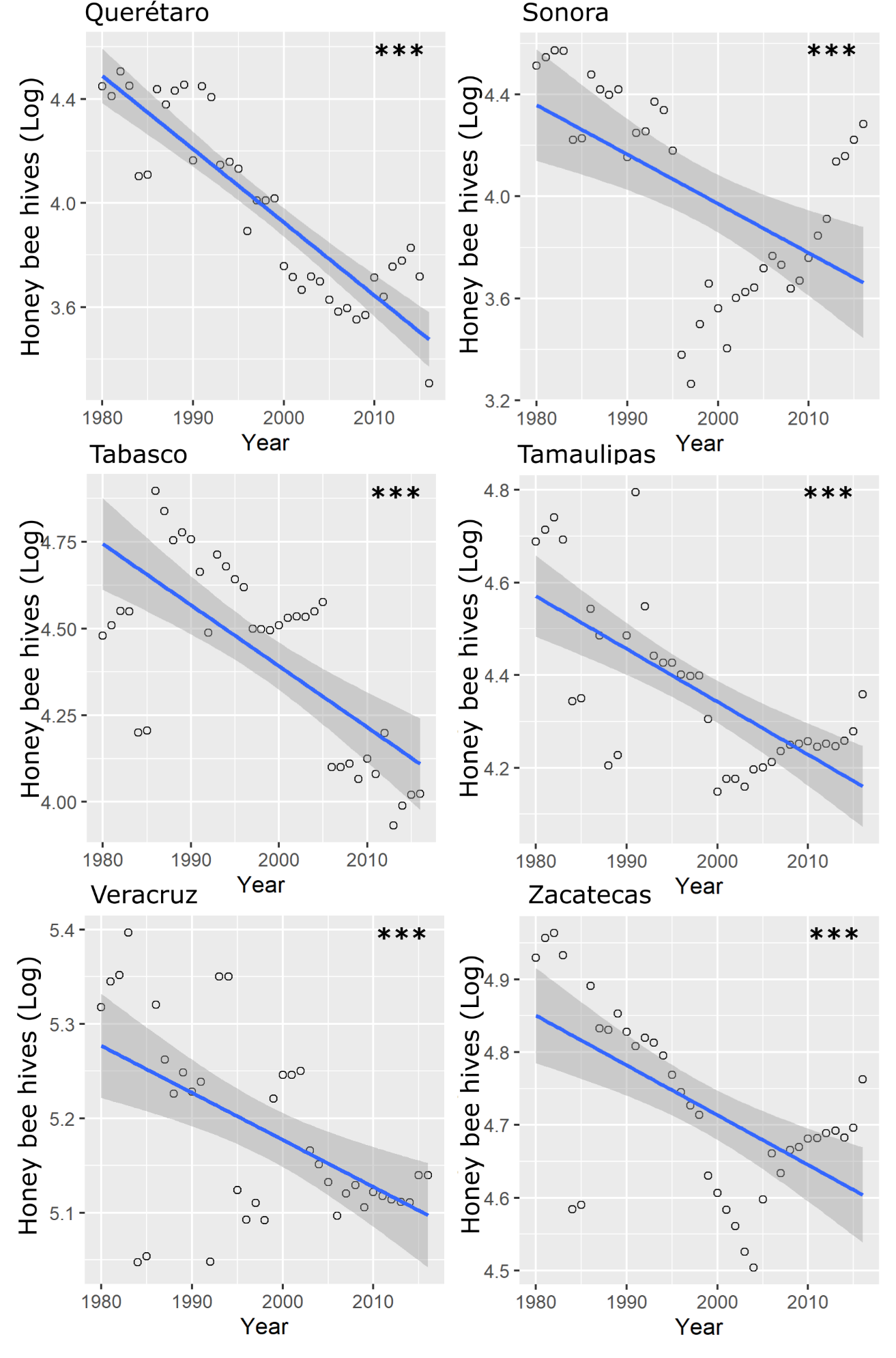


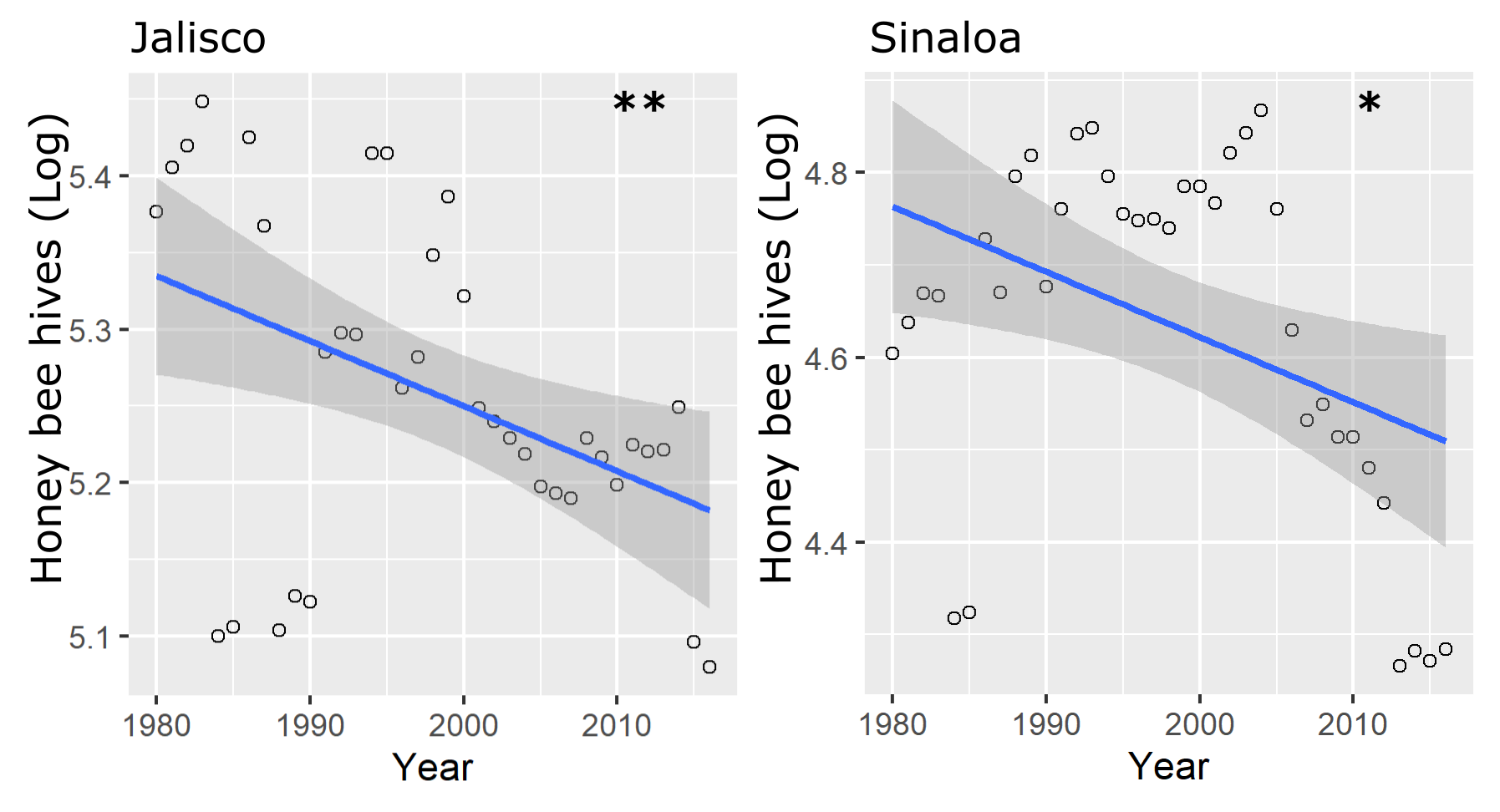


**Figure S1.** Simple linear regression models of a log 10-transformed number of hives as a dependent variable and year as an explanatory variable of states with significant decline effects (Table S1).


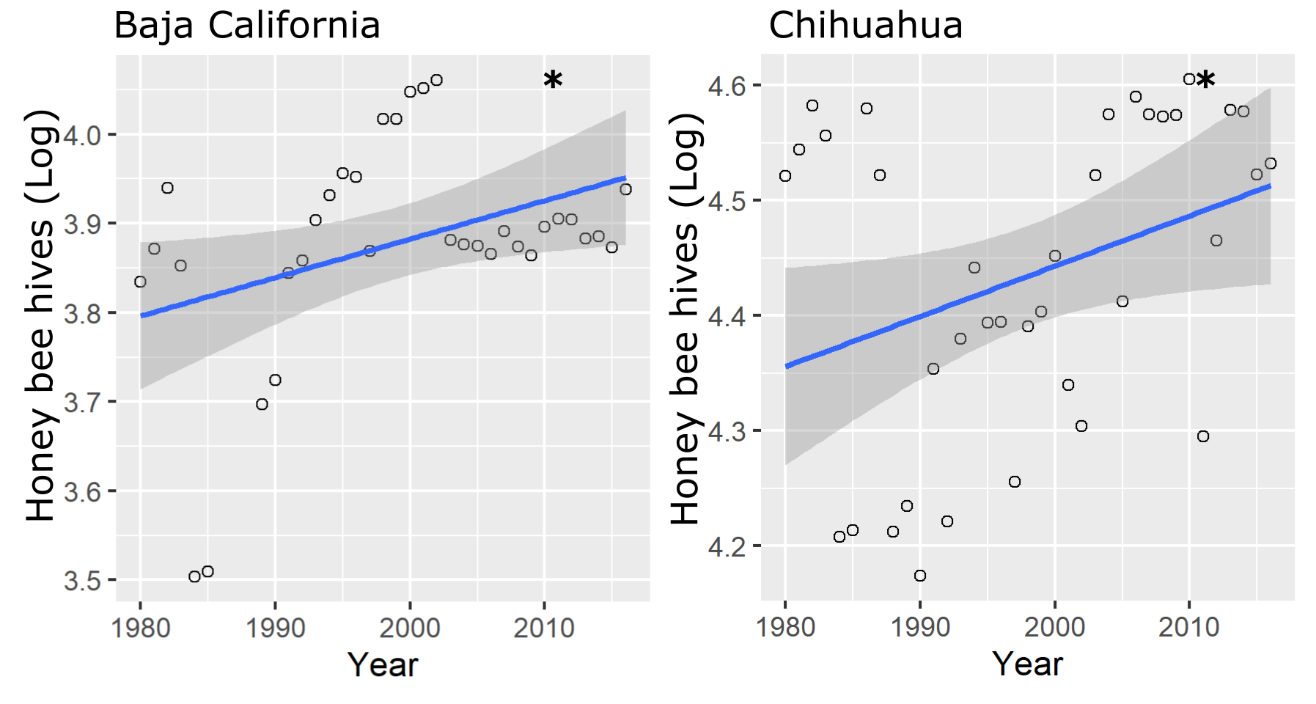


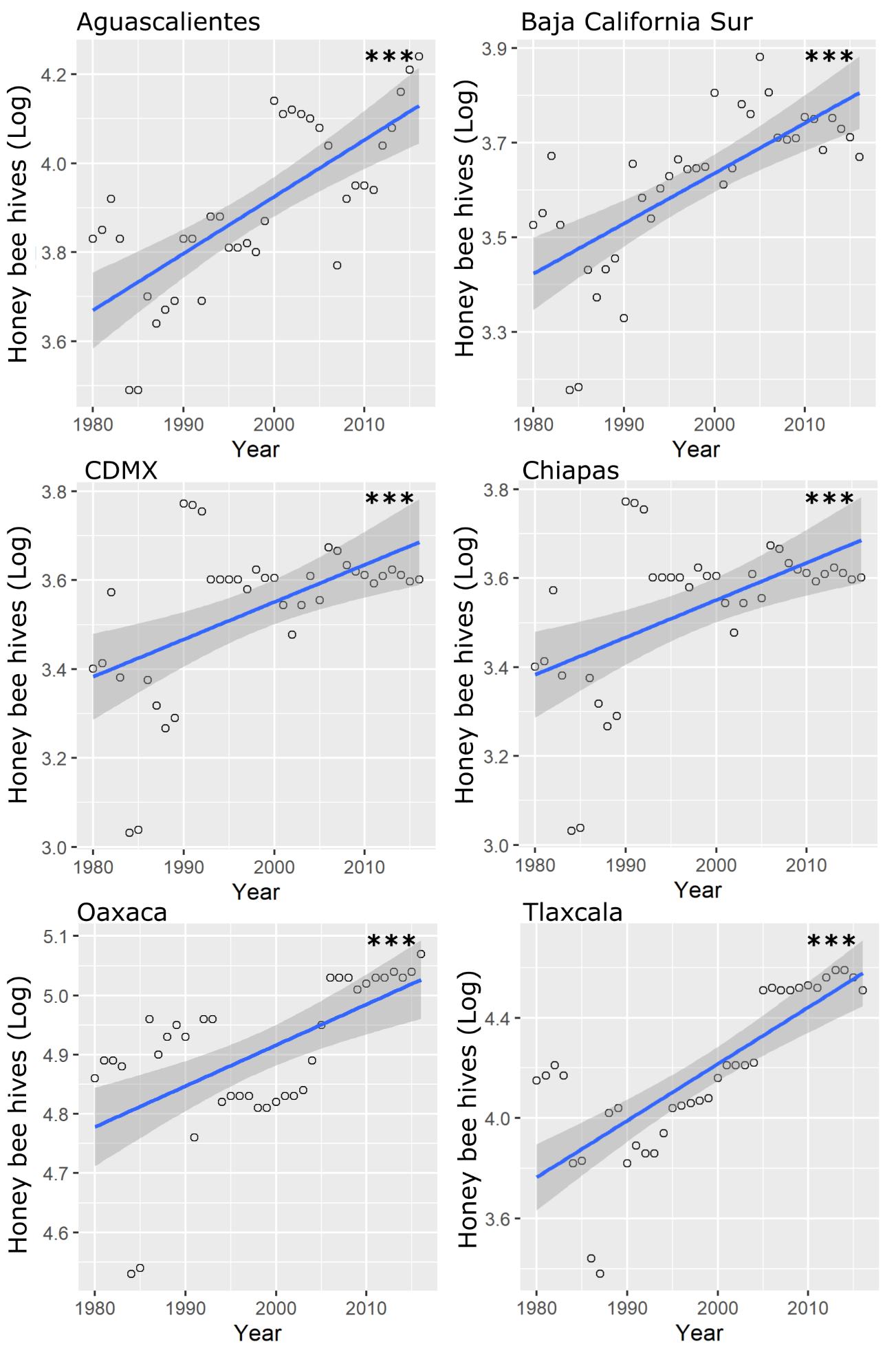


**Figure S2.** Simple linear regression models of a log 10-transformed number of hives as a dependent variable and year as an explanatory variable of states with significant increases (Table S1).
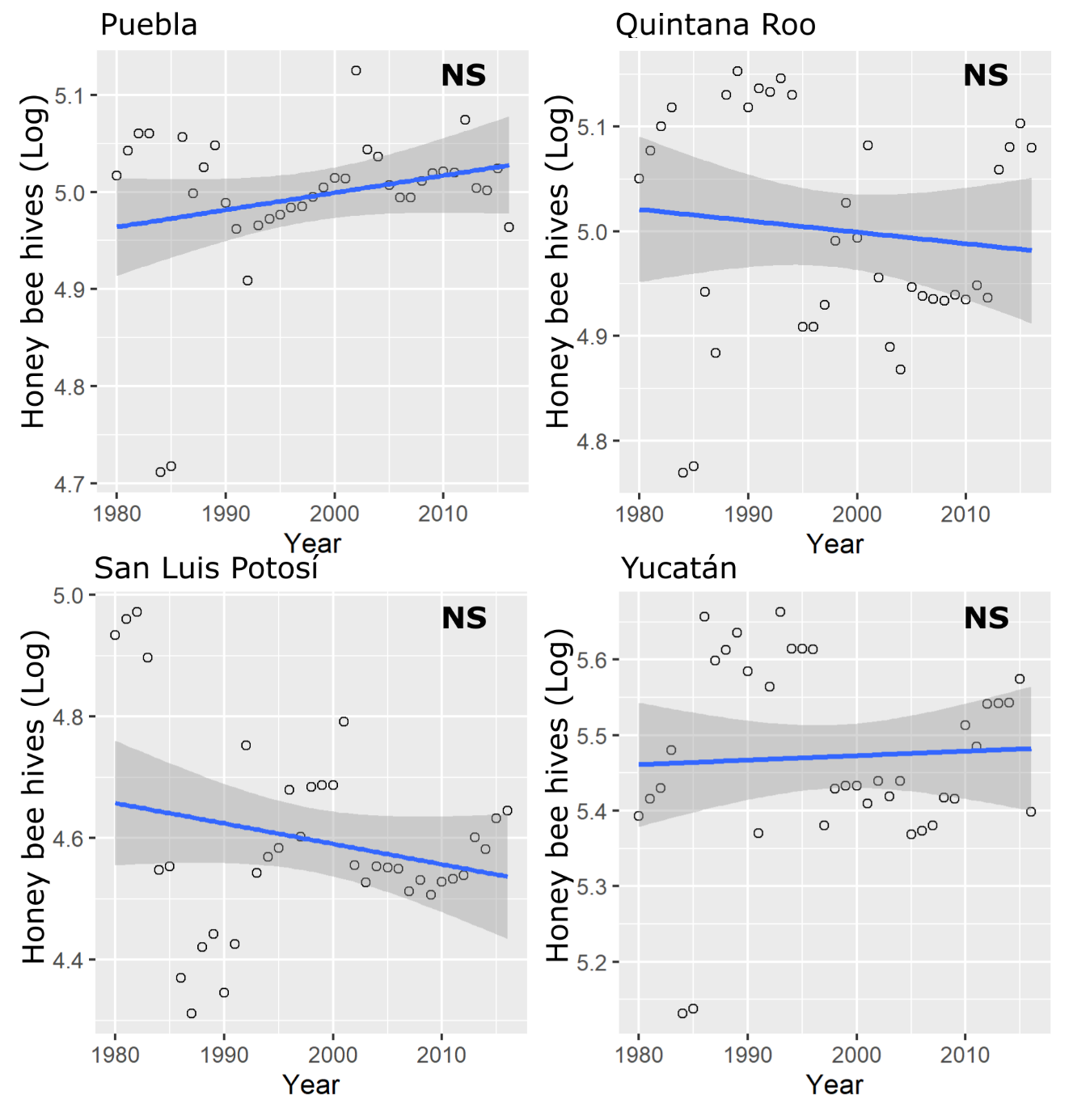


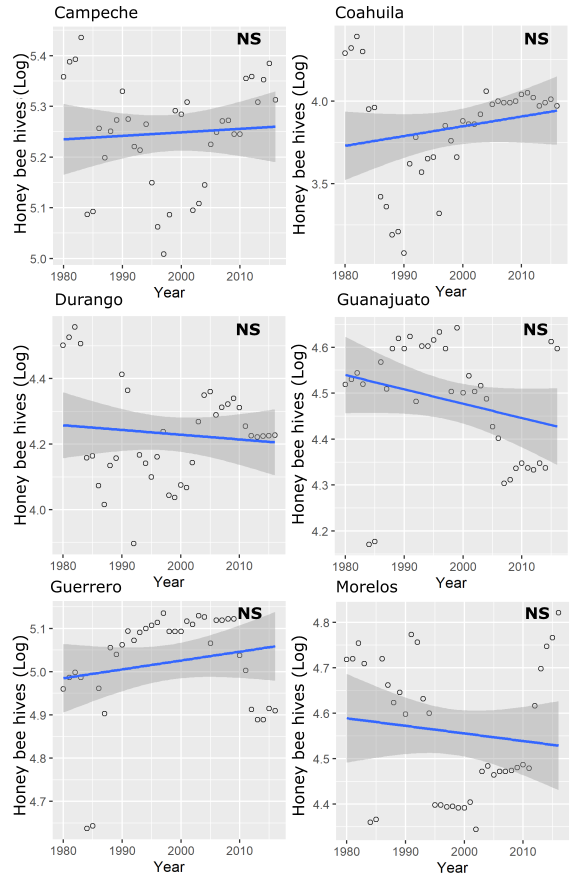


**Figure S3.** Simple linear regression models of a log 10-transformed number of hives as a dependent variable and year as an explanatory variable of states with non-significant trends (Table S1).


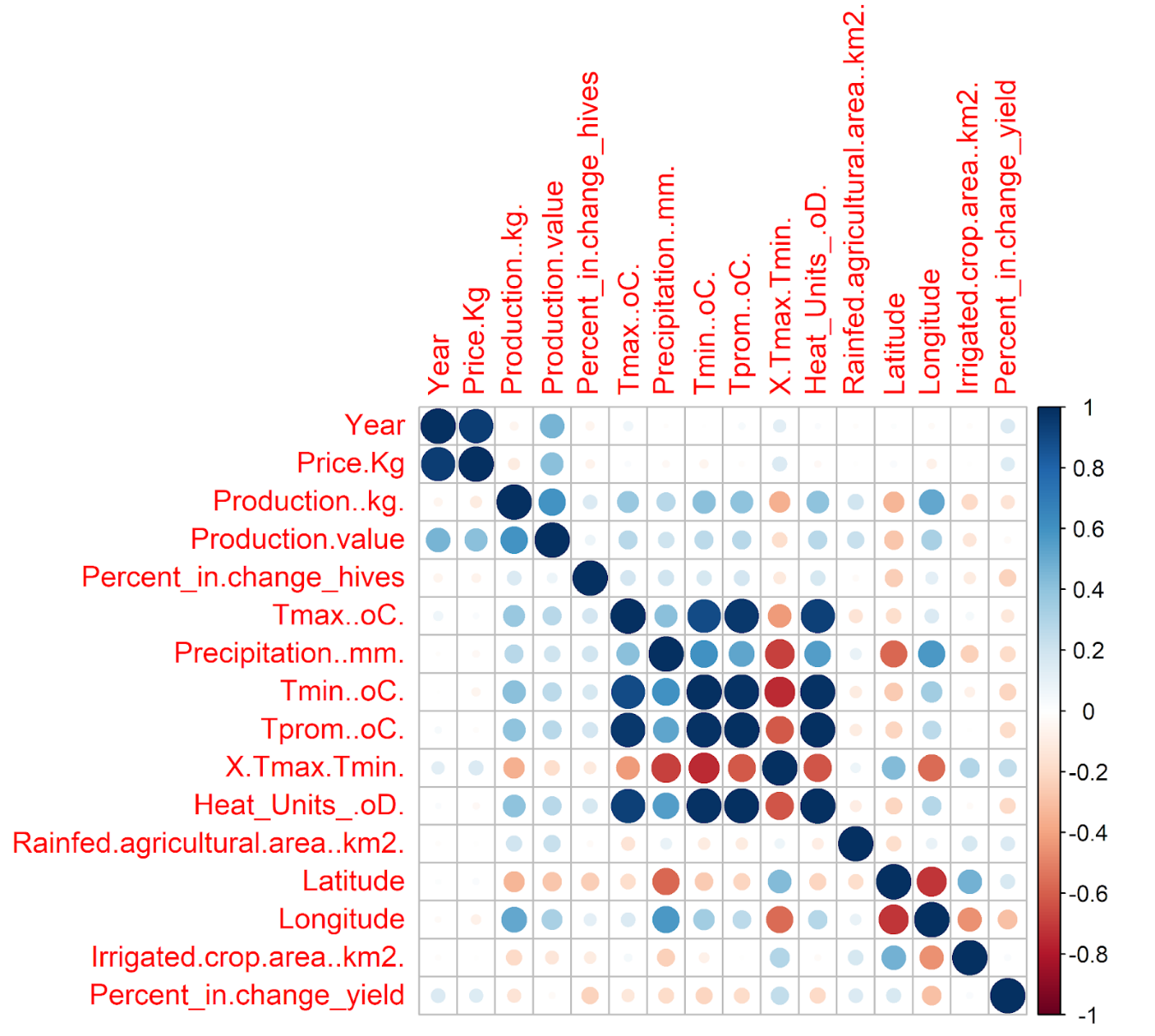


**Figure S4**. Pearson’s correlation coefficient for each of the potential predictors of GAMMs models.

**Model selection # Percent_change_hives**

lm <- lme((Percent_in.change_hives ~

Production..kg. +

Production.value +

Price.Kg +

Poverty.rate +

Year +

Tmax..oC.+

Tmin..oC. +

Rainfed.agricultural.area..km2.+

Irrigated.crop.area..km2. +

Precipitation..mm. ,

data = datos, random=list(Estado=pdIdent(~1)),

method = "ML")

glm <- lmer(Percent_in.change_hives ~

Production..kg. +

Production.value +

Price.Kg +

Poverty.rate +

Year +

Tmax..oC.+

Tmin..oC. +

Rainfed.agricultural.area..km2.+

Irrigated.crop.area..km2. +

Precipitation..mm. + (1|Estado),

data = datos, REML= FALSE)

gam <- gam(Percent_in.change_hives ~

s(Production..kg., bs="cs") +

s(Production.value, bs="cs") +

s(Price.Kg, bs="cs") +

Poverty.rate +

s(Year, bs="cs") +

te(Tmax..oC.,Tmin..oC., bs="ps") +

te(Rainfed.agricultural.area..km2.,Irrigated.crop.area..km2., bs="ps") +

s(Precipitation..mm., bs="cs") ,

data = datos,

correlation=corAR1(form=~1|Estado), method = "ML"

gamm <- gamm(Percent_in.change_hives ~

s(Production..kg., bs="cs") +

s(Production.value, bs="cs") +

s(Price.Kg, bs="cs") +

Poverty.rate +

s(Year, bs="cs") +

te(Tmax..oC.,Tmin..oC., bs="ps") +

te(Rainfed.agricultural.area..km2.,Irrigated.crop.area..km2., bs="ps") +

s(Precipitation..mm., bs="cs") ,

data = datos,

correlation=corAR1(form=~1|Estado),

method = "REML")

Model comparation

AIC(lm,glm,gam,gamm$lme)

df AIC

lm 14.00000 748.03732

glm 14.00000 748.03732

gam 59.07013 687.09615

gamm$lme 20.00000 -76.53363

Best model gamm AIC **-76.53363**

**Model selection # Honey_yield**

Lm2 <- lme((Percent_in.change_yield ~

Production..kg. +

Production.value +

Price.Kg +

Poverty.rate +

Year +

Tmax..oC.+

Tmin..oC. +

Rainfed.agricultural.area..km2.+

Irrigated.crop.area..km2. +

Precipitation..mm. ,

data = datos, random=list(Estado=pdIdent(~1)),

method = "ML")

glm2 <- lmer(Percent_in.change_yield ~

Production..kg. +

Production.value +

Price.Kg +

Poverty.rate +

Year +

Tmax..oC.+

Tmin..oC. +

Rainfed.agricultural.area..km2.+

Irrigated.crop.area..km2. +

Precipitation..mm. + (1|Estado),

data = datos, REML= FALSE)

gam2 <- gam(Percent_in.change_yield ~

s(Production..kg., bs="cs") +

s(Production.value, bs="cs") +

s(Price.Kg, bs="cs") +

Poverty.rate +

s(Year, bs="cs") +

te(Tmax..oC.,Tmin..oC., bs="ps") +

te(Rainfed.agricultural.area..km2.,Irrigated.crop.area..km2., bs="ps") +

s(Precipitation..mm., bs="cs") ,

data = datos,

correlation=corAR1(form=~1|Estado), method = "ML"

gamm2 <- gamm(Percent_in.change_yield ~

s(Production..kg., bs="cs") +

s(Production.value, bs="cs") +

s(Price.Kg, bs="cs") +

Poverty.rate +

s(Year, bs="cs") +

te(Tmax..oC.,Tmin..oC., bs="ps") +

te(Rainfed.agricultural.area..km2.,Irrigated.crop.area..km2., bs="ps") +

s(Precipitation..mm., bs="cs") ,

data = datos,

correlation=corAR1(form=~1|Estado),

method = "REML")

Model comparation

AIC(lm2,glm2,gam2,gamm2$lme)

df AIC

lm2 14.00000 1380.394

glm2 14.00000 1380.394

gam2 53.66473 1472.353

gamm2$lme 20.00000 1214.937

Best model gamm AIC **1214.937**


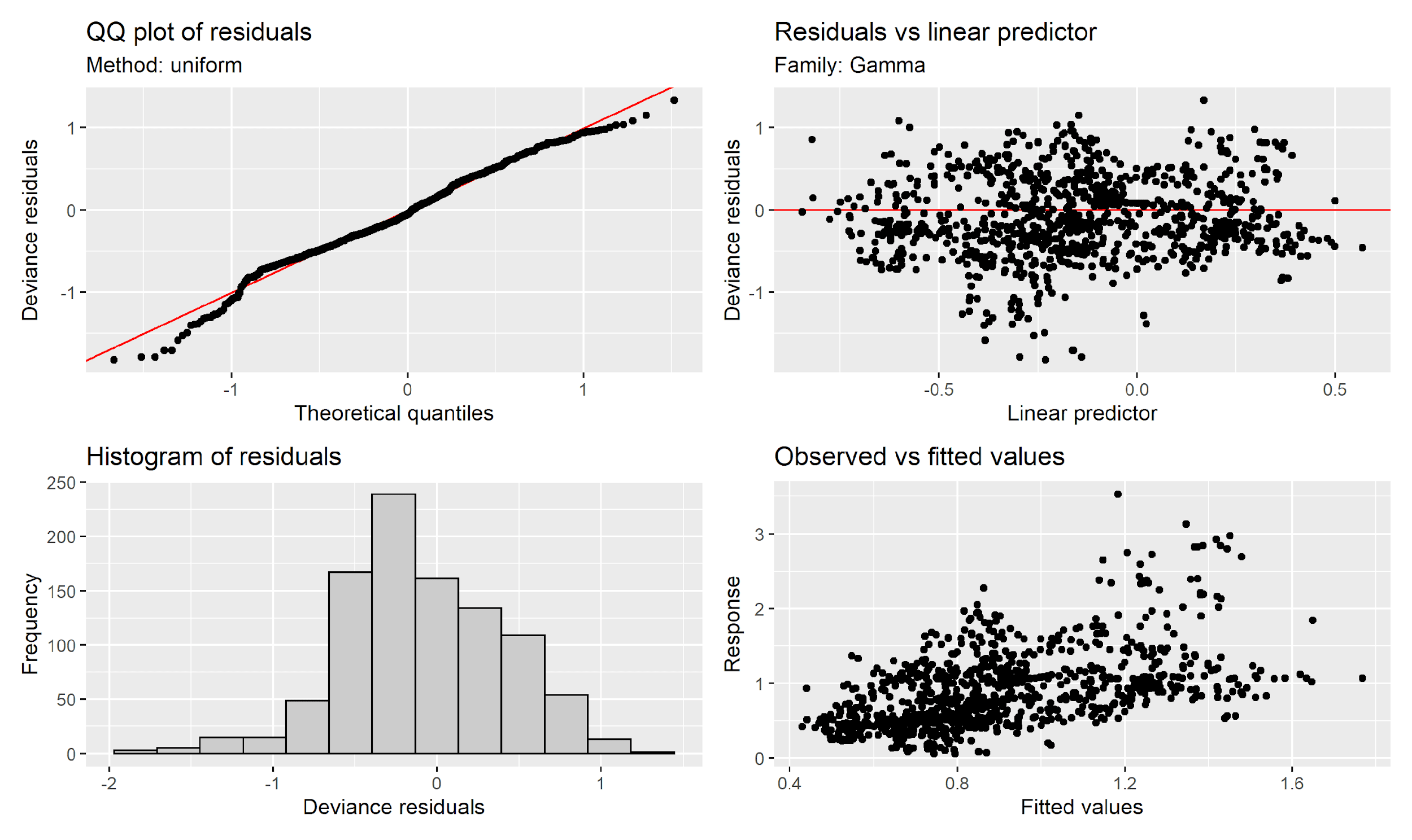


**Figure S5.** Graphical validation tools for the GAMM analysis of the percent change in the number of honey bee hives. The QQ-plot and the histogram are used to assess normality and the residuals versus fitted values homogeneity. The response against fitted values should ideally show a straight line.


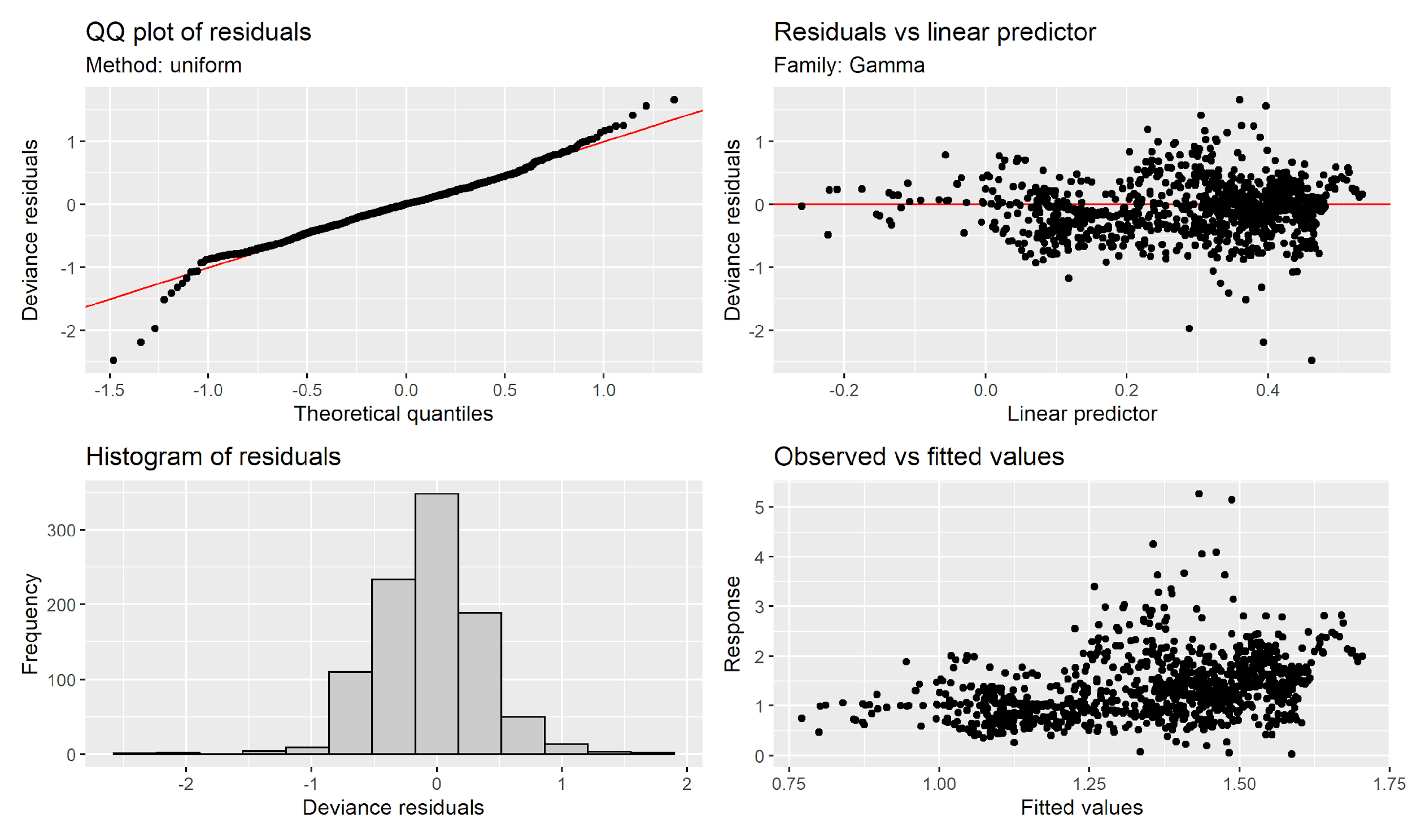


**Figure S6.** Graphical validation tools for the GAMM analysis of the percent change in yield of honey. The QQ-plot and the histogram are used to assess normality and the residuals versus fitted values homogeneity. The response against fitted values should ideally show a straight line.
